# Development of a kidney microphysiological system hardware platform for microgravity studies

**DOI:** 10.1038/s41526-024-00398-0

**Published:** 2024-05-11

**Authors:** Kendan A. Jones-Isaac, Kevin A. Lidberg, Catherine K. Yeung, Jade Yang, Jacelyn Bain, Micaela Ruiz, Greta Koenig, Paul Koenig, Stefanie Countryman, Jonathan Himmelfarb, Edward J. Kelly

**Affiliations:** 1 Department of Pharmaceutics, University of Washington, Seattle, WA USA; 2 Department of Pharmacy, University of Washington, Seattle, WA USA; 3 Kidney Research Institute, Seattle, WA USA; 4 BioServe Space Technologies, University of Colorado, Boulder, CO USA; 5 Present Address: RayzeBio, San Diego, CA USA

**Keywords:** Translational research, Medical research, Lab-on-a-chip

## Abstract

Determining the physiological effects of microgravity on the human kidney is limited to relatively insensitive tests of biofluids (blood and urine) that do not return abnormal results until more than 50% of kidney function is lost. We have developed an “organ on chip” microphysiological model of the human kidney proximal tubule (PT-MPS) that can recapitulate many kidney functions and disease states and could play a critical role in determining mechanisms of early kidney dysfunction in microgravity. However, the ground-based PT-MPS system is incompatible with spaceflight as it requires a large pneumatic system coupled to a cell incubator for perfusion and intensive hand-on manipulation. Herein, we report the hardware engineering and performance of the Kidney Chip Perfusion Platform (KCPP), a small, advanced, semi-autonomous hardware platform to support kidney microphysiological model experiments in microgravity. The KCPP is composed of five components, the kidney MPS, the MPS housing and valve block, media cassettes, fixative cassettes, and the programable precision syringe pump. The system has been deployed twice to the ISSNL (aboard CRS-17 and CRS-22). From each set of ISSNL experiments and ground-based controls, we were able to recover PT-MPS effluent for biomarker analysis and RNA suitable for transcriptomics analysis demonstrating the usability and functionality of the KCPP.

## Introduction

The microgravity environment induces a plethora of pathophysiological changes that resemble accelerated aging including wasting of skeletal muscle^1^, bone demineralization^2^, and metabolic and cardiovascular dysregulation^3–5^. In the case of bone mineral homeostasis, the kidneys control the excretion and retention of calcium, phosphate, and other essential ions^6^. The kidneys are also responsible for the generation of the active form of vitamin D, 1α,25-(OH)_2_ vitamin D_3_, which plays a critical role in a multitude of biological functions including bone health^7^.

Directly evaluating the impact of microgravity on kidney function at the molecular and cellular level is not feasible in astronauts due to the inherent risk of performing invasive kidney biopsies^8^. While studies can be conducted in rodents, the results may not truly reflect changes occurring in humans. To address the question of how microgravity affects human physiology, the National Center for Advancing Translational Sciences (NCATS) at the National Institutes of Health created the “Tissue Chips in Space” program that leveraged tissue engineering platforms to recapitulate human physiology in the environment of space. Selected research teams were funded to each send two projects to the International Space Station National Lab (ISSNL) during the four-year funding period.

Microfluidic-based microphysiologic systems (MPS) represent an advancement in cell culture techniques aimed at better replicating the tissue-specific in vivo environment. We have previously reported the development of an MPS-based model of the kidney proximal tubule (PT-MPS) utilizing a commercially available platform developed by Nortis Inc^9^. The Nortis™ system is designed for use with a tubing-free pneumatic-driven pump system but requires a substantial footprint, presents logistical challenges within the lab, and is not suitable in all research contexts. Therefore, the Kidney Chip Perfusion Platform (KCPP), a piston-based device, was developed to support an MPS-based kidney proximal tubule model.

The PT-MPS has been used to study a variety of disease states (e.g., aristolochic acid nephropathy and proteinuria)^10,11^ and the responses to drug/xenobiotic-induced kidney injury^12–14^. In addition, the robustness of this system was independently tested in collaboration with the NCATS-funded Tissue Chips Testing Centers^15,16^. To test the premise that microgravity is an accelerated environment for aging/disease progression, we evaluated proteinuria, kidney vitamin D metabolism, and nephrolithiasis (kidney stone disease)^17^. Herein we report the hardware engineering and performance of the KCPP in support of two missions to the ISSNL; the scientific results of the microgravity experiments are presented in an accompanying manuscript.

## Results

### Kidney chip perfusion platform system

**Fig. 1:**
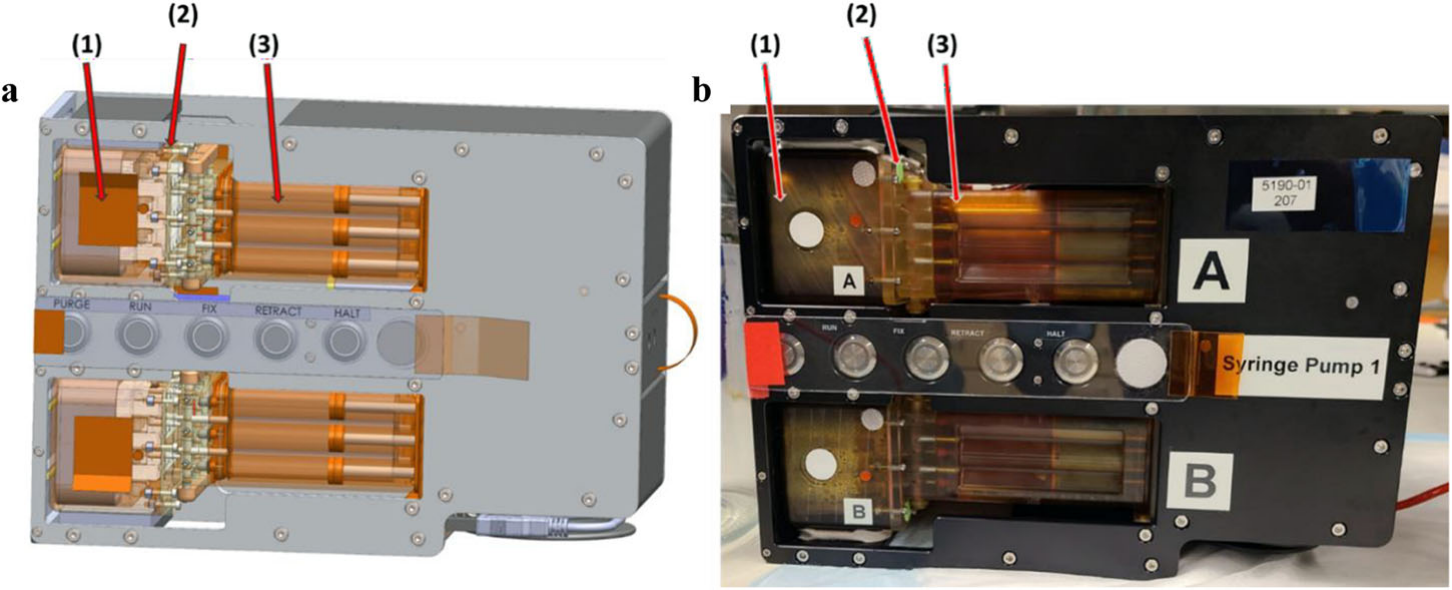
Schematic and realization of KCPP hardware. **a** Schematic and real life **b** view of the novel KCPP programmable perfusion platform showing six Triplex chips (1) situated within a housing unit after integration into the adapter unit (2) and media cassette (3).

Our team developed the KCPP hardware, addressed NASA safety and regulatory requirements, and facilitated the transition to a spaceflight certified and capable system. The platform is composed of five components, the kidney MPS, the MPS housing and valve block, media cassettes, fixative cassettes, and the programable precision syringe pump. Each KCPP (Fig. 1) is comprised of over 2500 custom-designed and machined components. In a ground-based lab, preparing and assembling these components is a lengthy process and requires sustained, active engagement; the crew aboard the ISS have a limited number of hours to conduct scientific experiments and operate on a strict schedule. The innovation of the KCPP over the in-lab process is a dramatic reduction in complexity and time commitment. For example, in the lab, switching between maintenance and experimental media requires several hours. This process was simplified with a pump interfacing to the MPS housing and valve block which can accept pre-loaded media or fixative cassettes.

The pump provides a continuous flow of media or fixative while maintaining temperature control at 37^o^ C. The pump uses a stepper motor to provide translation of a carriage which simultaneously depresses 18 syringe plungers. Preloading the media and fixative cassettes during the final pre-launch preparation phase streamlines the on-orbit protocol followed by the assigned astronaut on board the ISSNL. Additionally, the software for the pump only requires 5 operating modes for the experiment: “Purge” initiates the pump to engage the syringe pistons and prime the channels connecting the cassettes and the MPS, “Run” initiates perfusion with media at 0.5 µL per min, “Fix” perfuses fixative at 10 µL per min, “Retract” resets the pump plunger positions for sample housing and valve block and media/fixative cassette removal, and “Halt” stops all piston movement. The perfusion rate for media and fixation is programmable and readily modified. Thus, while the KCPP is a complex work of engineering, the interface for users on the ISSNL is intuitive and user-friendly.

Media can be loaded into nine individual channels separated by effluent bag cavities within a single media cassette. The media is contained in the channels between an O-ring piston and a septum. A cannula from the valve block pierces the septa when installed and allows the piston to push media into the PT-MPS. The media flows through the PT-MPS and is collected in effluent bags that are sealed with septa that are also pierced by cannula. The effluent bag cavities have containment plugs with O-rings on a retention plate. The waste media fills the effluent bags and is contained for post-flight analysis.

### KCPP Space Reduction Advancements

**Fig. 2:**
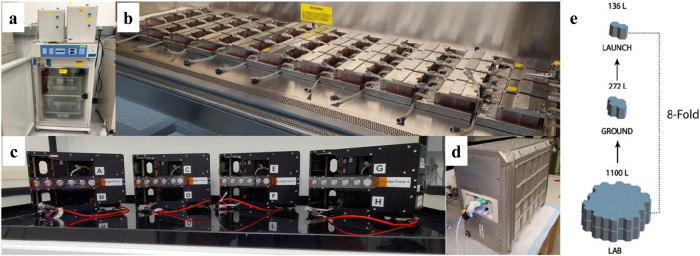
Reduction of chip experimental footprint. **a** Image depicting space 24 Triplex Chips occupy when the platforms and shelving are placed with the docking stations within tissue culture incubators, with the pneumatic pumps mounted on the incubator, **b** image depicting laboratory space that 44 Nortis Triplex Chips occupy when attached to platforms and shelves in a six-foot biosafety cabinet, **c** image of KCPP programmable perfusion platforms- four platforms are capable of perfusing 24 Nortis Triplex Chips, **d** powered locker used in the Kidney Chips experiment supplies power to four KCPP pumps, **e** KCPP platform reduces the space required to perfuse 48 Nortis Triplex Chips by 8-fold enabling astronauts to work within the limited space of the Life Sciences Glovebox. [2X incubators + 1X CO_2_ Tank = 1100 L Each Locker measures ~68 L (67.3 L)].

Although the overall footprint of an individual PT-MPS in the lab is small, the specialized equipment required to perfuse the devices is quite large. As shown in Fig. 2a, b, the individual components required to run experiments in a conventional laboratory require an entire tissue culture incubator. The availability of space on ISSNL is limited but the KCPP reduces the required footprint 8-fold (1100 L to 136 L) allowing 24 PT-MPS to be housed and perfused within the locker space allocated to our group on board the ISSNL (Fig. 2c–e). We have previously used commercially available syringe pumps to run PT-MPS experiments; 24 PT-MPS require eight syringe pumps to independently perfuse each of the 72 PT-MPS tubules. As shown in Supplementary Fig. 1, the ground-based system accommodates two pumps per tissue culture incubator, necessitating four separate incubators for 24 PT-MPS. In addition to significant space reductions from the KCPP, we have also eliminated the use of tubing, as the PT-MPS directly interfaces with the media blocks in the valve assembly. With syringe pumps, each individual PT-MPS tubule requires approximately 1 meter of tubing to connect media syringes outside of the incubators with PT-MPS within the incubator. Thus, in addition to creating a simple user-interface for operation on the ISSNL, the KCPP exponentially shrinks the footprint requirements compared to conventional terrestrial PT-MPS experiments.

### Testing and validation of the KCPP

**Fig. 3:**
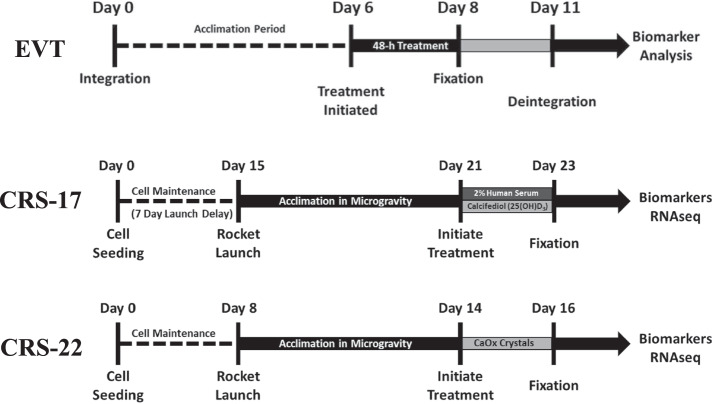
Experimental timeline for engineering validation test (EVT), CRS-17, and CRS-22. Experimental timelines for ground-based EVT and flight-based CRS-17 and CRS-22, showing timeframes for pre-launch, acclimation period, and exposure period.

An experiment validation test (EVT) was performed prior to each launch to assess the ability of the perfusion platform to maintain kidney PT-MPS cultures over the duration of the proposed experiments (Fig. 3). Kidney PT-MPS were loaded into the MPS housing and then integrated with the valve block and then into the perfusion platform. The devices were then cultured for six days in maintenance media to simulate a period of acclimation to microgravity. On day six, maintenance media cassettes were exchanged for treatment media cassettes and perfusion was continued for a 48-h treatment phase. On day eight, treatment media cassettes were removed and exchanged for fixative cassettes containing either RNAlater® or formalin. The effluent from both the maintenance and treatment media were stored at −80 °C for later analysis. Once the fixative cassette was installed, the fixative/preservative was perfused for 1 hour after which the platform components were deintegrated and the PT-MPS were stored at −80 °C or 4 °C for later analysis.

**Fig. 4:**
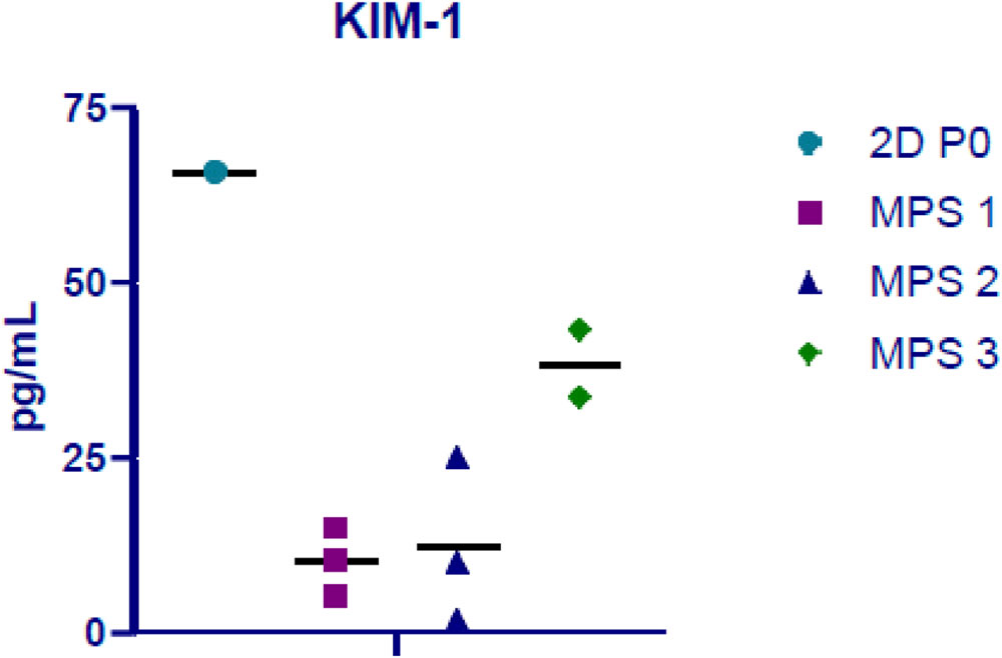
KIM-1 levels in effluents of kidney MPS cultured for 11 days in the KCPP and in 2D cultured PTECs at passage 0 after 7 days. 2D P0 sample=control cells at passage 0 cultured in 2D, MPS 1, 2, and 3=PT-MPS devices cultured in 3D with cells from 3 kidney cell donors.

Kidney Injury Molecule-1 (KIM-1), a protein secreted into the urinary filtrate by the proximal tubule, served as a biomarker of cell stability in the PT-MPS during EVT; we measured the secretion of KIM-1 in effluents. We have previously shown that basal secretion of KIM-1 in PT-MPS is low but is markedly increased in response to nephrotoxic insults^14–16^. As shown in Fig. 4, we observed low levels of KIM-1 from multiple PT-MPS evaluated in the EVT. For reference, a sample of 2D PTEC culture supernatant was included, but it should be noted that higher KIM-1 levels are expected in 2D cultures due to the cells being in a persistent proliferative state while PTECs cultured in MPS devices are relatively quiescent^15^.

### KCPP System Performance

To date, we have completed two launches of the KCPP system to the ISSNL. The first launched on board SpaceX Commercial Resupply Services mission 17 (CRS-17) and the second on SpaceX CRS-22. On the CRS-17, we evaluated vitamin D metabolism and proteinuric responses, and on CRS-22, we studied a calcium oxalate microcrystal model of nephrolithiasis. To assess the overall performance of the KCPP hardware, we evaluated the ability to recover PT-MPS effluents for biomarker analyses as well as successful perfusion of RNAlater™ and recovery of high-quality RNA for gene expression studies. The basic study design and timelines for CRS-17 and CRS-22 are shown in Fig. 3. Each launch consisted of 24 PT-MPS in-flight (microgravity) with a matched cohort of 24 ground-based PT-MPS. The CRS-17 launch consisted of 4 different PTEC donors (two males & two females) while CRS-22 included 6 different donors (three males & three females). The number of samples obtained for RNAseq analysis is shown in Table 1 for CRS-17 and -22, while Table 2 shows a similar breakdown for effluent retrievals for CRS-17 and -22.

**Table 1:** PT-MPS tubules available for RNAseq analysis listed according to launch mission, test environment, test articles, and individual donor ID

CRS-17		Donor	M1	M2	F1	F2		
	Flight (33/54, 61%)							
	Media		3/6	3/3	4/6	0/3		
	Vitamin D		4/6	0/3	2/6	2/3		
	2% Human serum		6/6	2/3	5/6	2/3		
	Ground (39/54, 72%)							
	Media		2/6	0/3	3/6	3/3		
	Vitamin D		5/6	3/3	6/6	2/3		
	2% Human serum		5/6	2/3	6/6	2/3		

CRS-22		Donor	F1	F2	F3	M1	M2	M3
	Flight (70/72, 97%)							
	Media		3/3	6/6	3/3	3/3	3/3	6/6
	Vitamin D		3/3	6/6	3/3	3/3	3/3	5/6
	2% Human serum		3/3	5/6	3/3	3/3	3/3	6/6
	Ground (70/72, 97%)							
	Media		3/3	6/6	3/3	3/3	3/3	6/6
	Vitamin D		3/3	6/6	3/3	3/3	3/3	6/6
	2% Human serum		3/3	6/6	3/3	1/3	3/3	6/6

In addition, the total % recovery of ground versus flight (microgravity) environments is listed. (M-male, F-female).

**Table 2:** PT-MPS tubules available for effluent biomarker analysis listed according to launch mission, test environment, test articles, and individual donor ID

CRS-17		Donor	M1	M2	F1	F2		
	Flight (349/72, 68%)							
	Media		6/9	7/9	10/12	0/6		
	Vitamin D		5/6	0/3	3/6	3/3		
	2% Human serum		5/6	2/3	6/6	2/3		
	Ground (48/72, 67%)							
	Media		2/9	0/9	5/12	6/6		
	Vitamin D		6/6	3/3	6/6	3/3		
	2% Human serum		5/6	3/3	6/6	3/3		

CRS-22		Donor	F1	F2	F3	M1	M2	M3
	Flight 63/72, 88%%)							
	Media		3/3	6/6	3/3	2/3	3/3	5/6
	Vitamin D		3/3	6/6	1/3	3/3	3/3	4/6
	2% Human serum		2/3	5/6	2/3	3/3	3/3	6/6
	Ground (67/72, 93%)							
	Media		3/3	5/3	3/3	2/3	3/3	6/6
	Vitamin D		3/3	5/6	2/3	3/3	2/3	6/6
	2% Human serum		3/3	6/6	3/3	3/3	3/3	6/6

In addition, total % recovery of ground versus flight (microgravity) environments is listed. (M-male, F-female).

### Cell viability and RNA stability

The criteria for determining a “high quality” sample for RNAseq was based on the ability to retrieve RNA from the PT-MPS tubules with a detergent solution, and subsequent total RNA isolation. Quality controls included Bioanalyzer™ RNA integrity determination, RNA concentrations as well as subsequent RNAseq analysis (data not shown) and reported in Table 3. The criterion for “usable” sample for effluent analysis was based on the retrieval of media in individual effluent bags after thawing of the KCPP media cassette blocks. It is worth noting the differences in the rates of “usable samples” between CRS-17 and CRS-22. In CRS-17, approximately 30% of the samples (RNAseq and effluents) were unusable for both flight and ground due to mold contamination of the PT-MPS. In contrast, nearly 100% of the samples were usable in CRS-22. The mold contamination observed in CRS-17 was not related to KCPP performance but was likely a consequence of multiple launch delays that necessitated greater handling/transport of the PT-MPS from standard cell culture incubators to launch lockers, an additional media exchange, and small amounts of residual media on the cell injection port on the PT-MPS. To mitigate these issues for CRS-22, we employed rigorous PT-MPS sanitizing protocols as well as applied a medical-grade silicone-based sealant (Silastic A®) over the PT-MPS cell injection ports. It is also worth noting that the issues with launch delays for CRS-17 did not occur with CRS-22.

**Table 3:** Summary of RNA yields and quality (RIN scores) from EVT conducted in preparation for CRS-22

Sample ID	Group	Treatment	Qubit [RNA] ng per µL	RIN
Chip 1 A	PT9-M	PTEC Media	39.87	9.2
Chip 2 A	PT9-M	PTEC Media	60.23	9.5
Chip 2 B	PT9-M	PTEC Media	56.02	9.9
Chip 2 C	PT9-M	PTEC Media	53.23	9.8
Chip 3 A	PT9-M	CaOx 1000 µg per mL	28.6	9.5
Chip 4 A	PT9-M	CaOx 1000 µg per mL	58.39	10
Chip 4 B	PT9-M	CaOx 1000 µg per mL	27.33	10
Chip 4 C	PT9-M	CaOx 1000 µg per mL	67	9.7
Chip 5 A	PT9-M	CaOx 1000 µg per mL + KCIT 250 µM	24.36	10
Chip 5 B	PT9-M	CaOx 1000 µg per mL + KCIT 250 µM	23.42	10
Chip 5 C	PT9-M	CaOx 1000 µg per mL + KCIT 250 µM	53.83	10
Chip 6 A	PT9-M	CaOx 1000 µg per mL + KCIT 250 µM	83.5	10
Chip 6 B	PT9-M	CaOx 1000 µg per mL + KCIT 250 µM	66	10
Chip 6 C	PT9-M	CaOx 1000 µg per mL + KCIT 250 µM	35.5	10
Chip 13 A	PT6-F	PTEC Media	164.58	10
Chip 13 B	PT6-F	PTEC Media	82.71	9.9
Chip 13 C	PT6-F	PTEC Media	156.12	10
Chip 14 A	PT6-F	PTEC Media	108.76	10
Chip 14 B	PT6-F	PTEC Media	31.58	9.5
Chip 14 C	PT6-F	PTEC Media	160.74	9.9
Chip 15 A	PT6-F	CaOx 1000 µg per mL	233.06	9.7
Chip 15 B	PT6-F	CaOx 1000 µg per mL	88.42	10
Chip 15 C	PT6-F	CaOx 1000 µg per mL	165.67	9.9
Chip 16 A	PT6-F	CaOx 1000 µg per mL	17.34	3.9
Chip 16 B	PT6-F	CaOx 1000 µg per mL	237.36	10
Chip 16 C	PT6-F	CaOx 1000 µg per mL	216.84	10
Chip 17 C	PT6-F	CaOx 1000 µg per mL + KCIT 250 µM	209.87	10
Chip 18 A	PT6-F	CaOx 1000 µg per mL + KCIT 250 µM	97.47	10
Chip 18 B	PT6-F	CaOx 1000 µg per mL + KCIT 250 µM	195.12	10
Chip 18 C	PT6-F	CaOx 1000 µg per mL + KCIT 250 µM	23.69	10

Samples are derived from two different PTEC donors (PT9-M and PT6-F) and grouped according to test articles (media control, calcium oxalate microcrystals [CaOx], or potassium citrate [KCIT].

## Discussion

KCPP is an integrated, automated, piston-based perfusion platform and enclosed cell culture environment designed to support MPS-based life sciences experimentation on board the ISSNL. Its compact design enables a significant reduction in the logistical challenges and spatial footprint required to implement these experiments aboard the confined space of the ISSNL. KCPP has been verified and space flight certified and is compliant with current NASA safety and interface requirements for space flight and use aboard the ISSNL. The system has been successfully utilized to support two space-based experimental studies designed to test the impact of microgravity on the function and pathophysiology of PTECs cultured in MPS aboard the ISSNL. In each instance, we were able to recover MPS effluent that could be analyzed for secreted biomarkers and RNA that was of sufficient quality for RNAseq bulk transcriptomics. Recovery of RNA demonstrates that cells remained viable and adherent throughout launch and orbit given that non-viable cells rapidly slough off and are swept out of the tubule. In addition, brightfield microscopy images of devices from platform testing phases confirm the presence of cells in the tubule with an epithelial phenotype^18^.

Biological data from the PT-MPS can uncover both major (viability, gross structural changes, injury, and proliferation) and subtle changes in cell functionality. The use of KIM-1 has been validated by the FDA^19^ as a urinary biomarker of nephrotoxicity—it is sensitive and easily measured without requiring termination of the experiment but does not provide mechanistic insight into the etiology of the damage. In contrast, RNAseq provides a snapshot of total cell transcriptomics and can identify subtle changes in homeostatic pathways (e.g., oxidative stress, cholesterol metabolism, drug metabolism, and transport). KIM-1 secretion and RNAseq analysis from CRS-17 are presented in a companion manuscript.

The improvement in sample recovery between missions emphasizes the importance of developing countermeasures against factors responsible for tubule or MPS device attrition. Looking to the future, extended studies using the KCPP system will facilitate the understanding of the long-term effects of spaceflight on renal physiology; to date, ground-based cultures of proximal tubule epithelia in the Nortis platform have remained viable and maintained an epithelial phenotype for more than 12 months with little maintenance other than twice-monthly media exchanges. We believe that extended experiments (6+ months) could provide insight into the effects of extended exposure to microgravity and/or space radiation and allow experiments that test kidney cell response to extended chemical exposures and the capacity of the kidney cells to recover from injury in microgravity.

While the KCPP functions nominally, there are limitations with both the KCPP and the kidney cell MPS. First, the KCPP still requires hands-on manipulation to exchange media cassettes- while the process is greatly accelerated compared with ground-based systems, required crew time could be prohibitive, especially for a 6+ month experiment. In principle, the capacity of the media cassettes could be increased to allow up to 1 month between media exchanges, but media stability at 37 °C for 30+ days has not been rigorously evaluated. In addition, transport and cold storage of several spare media cassettes may not be feasible due to space and weight limitations. Second, there is no real-time imaging capability for PT-MPS installed in the KCPP—removing a single PT-MPS from the sealed enclosure is difficult and could introduce contamination. More importantly, the lack of real-time image and data acquisition limits our abilities to modify or terminate experimental protocols in response to changes in cell morphology; improvements we hope to see developed in the future. Finally, the PT-MPS includes only a single cell type. While the proximal tubule epithelium is critical for the clearance of biological wastes, drugs, and environmental toxins, and is often subject to injury from toxic stimuli, it is only one of the 40+ cell types in the human kidney, all of which interact to create an intact organ^20^. Future advances in MPS and organoid technology are expected to include multiple types of kidney cells as well as vascular and immune components. We believe that continued development of a more autonomous perfusion platform and improved MPS technology can be used to identify sentinel events and mechanisms involved in the development of kidney injury and to predict and prevent kidney damage caused by spaceflight and long-term human space exploration.

## Methods

### KCPP Engineering

#### Chip housing & valve block

**Fig. 5:**
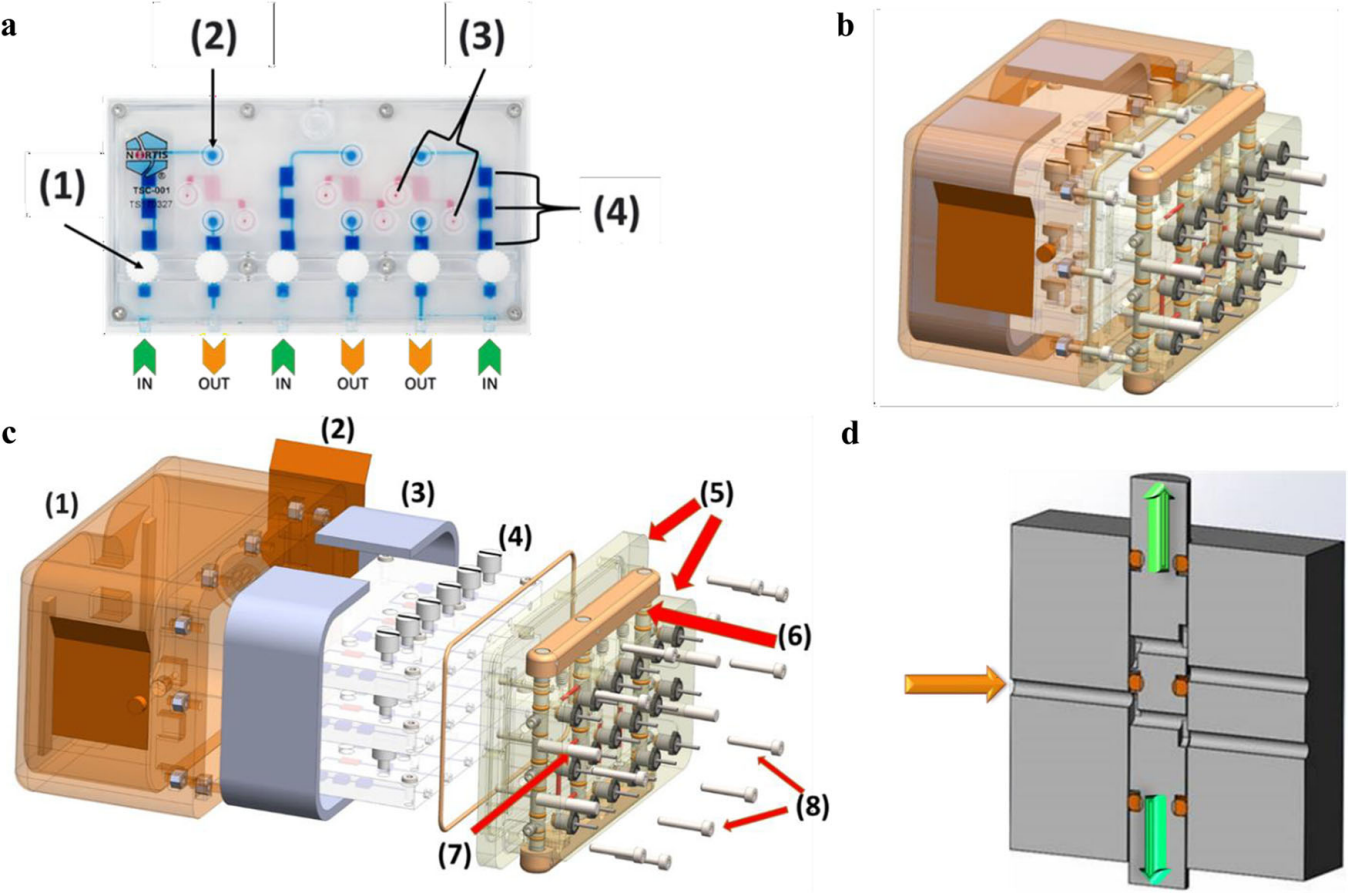
Nortis TCC, Triplex Housing & Valve Block Assembly. **a** Schematic depicting a Nortis Triplex Chip including; (1) port valve, (2) injection port, (3) matrix plug holes, (4) bubble traps, **b** integrated view of a Chip Housing and Valve Block Assembly, **c** exploded view of a Chip Housing & Valve Block Assembly including; (1) Triplex Housing, (2) Vent Cover, (3) Absorbent Padding, (4) Nortis Triplex Chips, (5) Valve Block, (6) Valve Bar, (7) Cannula, (8) Alignment Pins, and **d** diagram of the valve block assembly oriented such that media flows from left to right depicting the internal mechanism set to the purge configuration.

The MPS housing and valve block system is a protective sealed enclosure, designed with functions for purging bubbles during media or fixative cassette installation (Fig. 5). Considerable effort is taken in the lab to mitigate the risk of bubbles entering the MPS which leads to disruption of media flow and compromises the integrity of the PTEC lumen. Because of the unpredictable nature of air bubbles in microgravity, bubbles may bypass the series of buoyancy traps in the MPS on the ISS. The enclosure interfaces to the media cassette and fixative cassette via four alignment pins and 18 cannulas. The housing vents are sealed with two adhesive covers during launch operations to maintain a 5% CO_2_ and 100% humidity environment within the MPS housing. The valve block is designed with a valve bar system to direct flow through the valve block. Purging is performed when the valve bar is in the upper position as shown in Fig. 5d. When the valve bar is in purge mode, flow is diverted from the PT-MPS directly into the effluent bags. When the valve bar is in flow mode, flow is directed into the PT-MPS.

#### Media and Fixative Cassettes

**Fig. 6:**
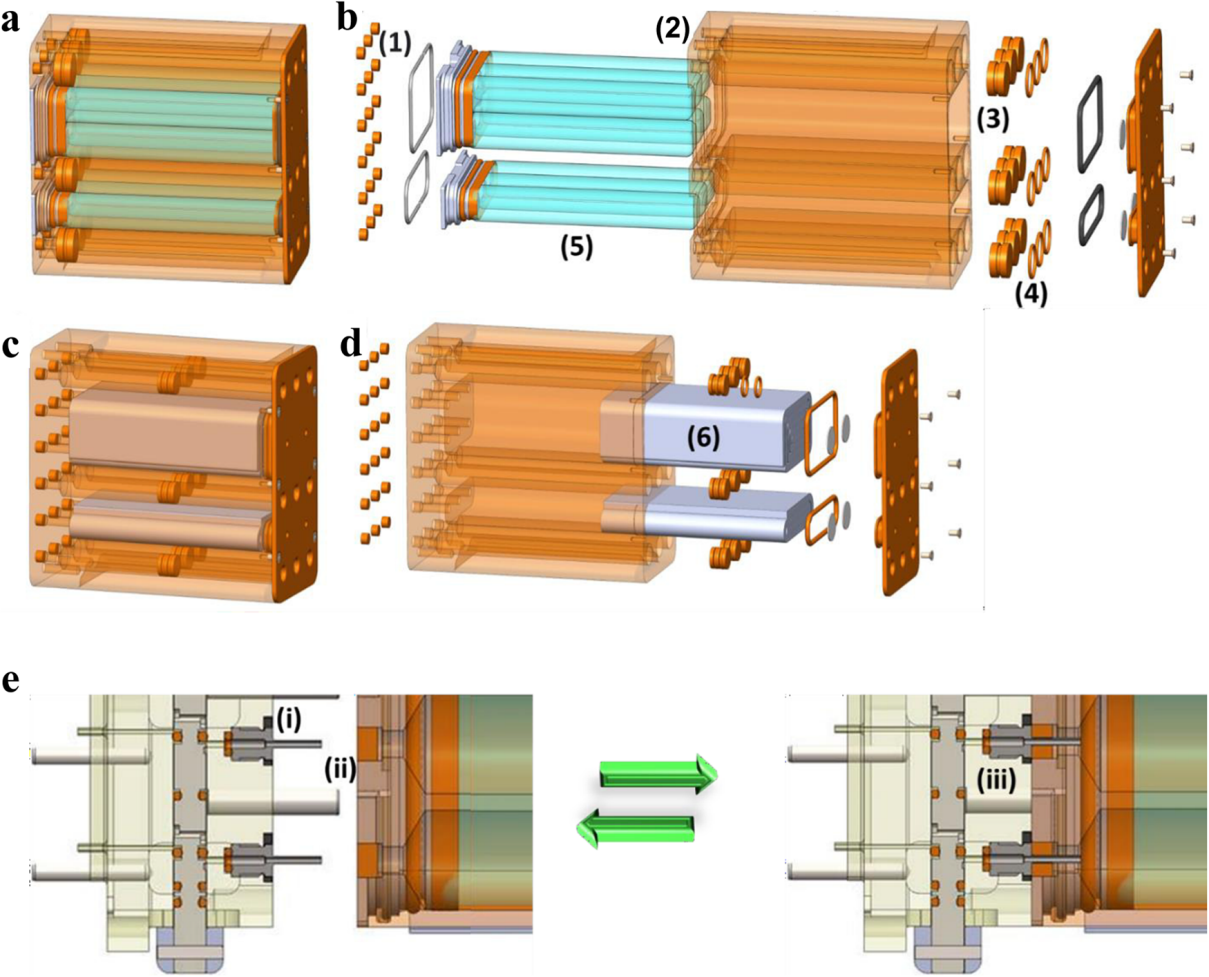
Media & Fixative Cassettes. **a** Integrated view of a media cassette assembly, **b** exploded view of a media cassette assembly, **c** integrated view of a fixative cassette assembly, **d** exploded view of a media cassette assembly (1) septa, (2) polyetherimide block, (3) piston, (4) piston O-ring, (5) FEP bags (media cassette only), (6) absorbent padding (fixative cassette only), **e** diagram depicting the self-sealing septa mechanism in separated (left) and connected (right) configurations.

The media cassette was designed to integrate directly with the chip housing and valve block and the KCPP to provide sufficient media to perfuse the PT-MPS for 10 days at a rate of 750 µL per day (Fig. 6a). Based on this flow rate (0.5 µL per min), the fluid shear stress (FSS) within the MPS tubule with a diameter between 100 µm and 120 µm is 0.47 dyn per cm^2^ – 0.82 dyn per cm^2^ (calculated by Poiseuille’s equation) is comparable to estimates of FSS in the human proximal tubule (0.7 dyn per cm^2^–1.2 dyn per cm^2^)^21^. The cassette consists of nine individual channels machined into an Ultem thermoplastic resin block. Each channel has a usable volume of 7.75 mL. The fluid is dispensed by mechanical plunger translation via the syringe pump. Once the fluid has passed out of the cassette and through the PT-MPS it returns to the housing and is stored in individually sealed bags in an adjacent chamber to the media channels. The fluid interfaces with the valve block via 18 cannulas piercing the corresponding septa in the bottom of the media cassette.

The sample effluent collection volume is sealed at the top of the cassette which provides an additional level of containment. When two levels of containment are required during cassette change-out operations, the KCPP system can be operated within the Microgravity Science Glovebox (MSG) or Life Science Glovebox (LSG) which provides an additional level of containment during crew manipulations of the media or fixative cassettes.

The fixative cassette is a modified version of the media cassette (Fig. 6c). The cassette provides fixative for the final stage of the experiment to preserve the cells in the PT-MPS. The cassette has nine individual channels machined into a block of Ultem. Each channel has a maximum volume of 3.8 ml. The fluid is dispensed via mechanical plunger translation via the syringe pump. Once the fluid has passed out of the cassette and through the PT-MPS it returns to the housing and is absorbed into layers of absorbent material in adjacent chambers to the fixative channels. The fluid interface to the valve block is via 18 cannulas piercing the corresponding septa in the bottom of the fixative cassette. The fixative cassette provides two levels of containment using O-rings on the pistons. Additional containment can be provided via outer bags, if needed.

#### KCPP Integration

**Fig. 7:**
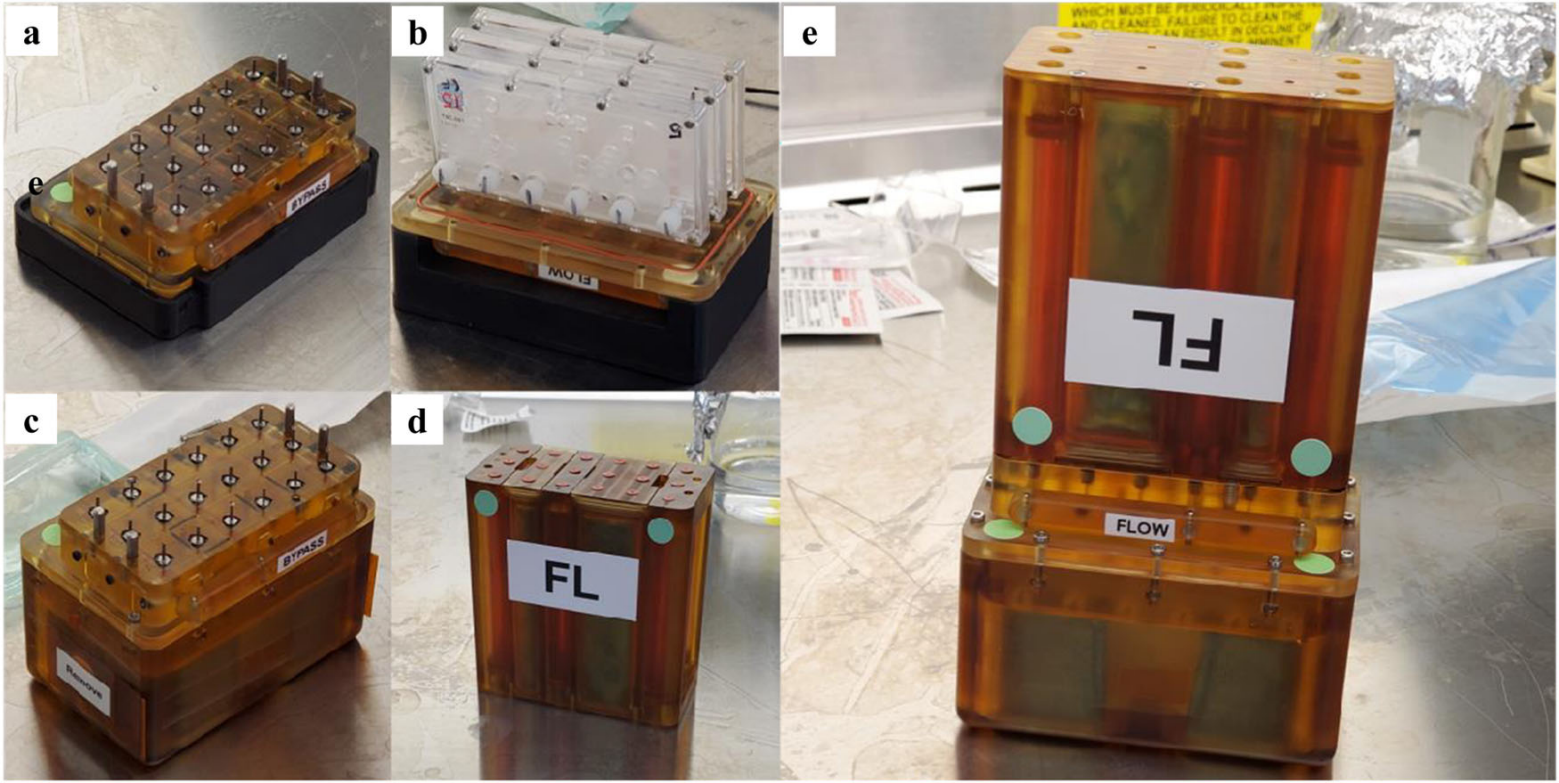
Stepwise integration of the chip environment. **a** Valve block, **b** three triplex chips integrated onto the valve block, **c** valve block & triplex housing integrated assembly, **d** launch media cassette, **e** media cassette, valve block & triplex housing.

The integration and assembly of the individual components of the KCPP are shown in Fig. 7. In brief, Fig. 7a–c depict a valve block, PT-MPS and integrated assembly, respectively. A media cassette is shown in Fig. 7d and all the assembled components are seen in Fig. 7e.

### MPS cell culture and bioanalytics

#### Tissue acquisition & cell culture

Whole human kidneys not suited for human transplantation were obtained from Novabiosis, Inc. (Research Triangle Park, NC) with all patient identifiers removed in accordance with a biospecimens procurement agreement. Primary human proximal tubule epithelial cells (PTECs) were isolated by mechanical and enzymatic dissociation and cultured as previously reported^22^. PTEC cultures were maintained serum-free in DMEM/F12 (Gibco, Grand Island, NY, Cat. #11330-032) supplemented with 1x insulin-transferrin-selenium-sodium pyruvate (ITS-A, Gibco, Cat. #51300044), 50 nM hydrocortisone (Sigma, St. Louis, MO, Cat. # H6909), and 1x Antibiotic-Antimycotic (Gibco, Cat. # 15240062). Upon reaching 75-80% confluence, PTECs were passaged by enzymatic digestion with 0.05% trypsin EDTA (Gibco, Cat. #25200056) and manual cell scraping to obtain a single-cell suspension which was subsequently neutralized with defined trypsin inhibitor/DTI (Gibco, Cat. #R007100) at a volume: volume ratio of 2:1 DTI: trypsin, then the cells were pelleted by centrifugation at 200 x *g* for 7 minutes, resuspended in maintenance media, and plated in cell culture treated flasks at >25% confluency (referred to as passage 1 or P1). For both EVTs and CRS-17/22 missions, media cassettes were loaded and then stored for 1 week at 4 °C before being warmed to 37 °C immediately prior to integration with the KCPP. At the end of the treatment duration, sample effluents were frozen at −80 °C.

#### Preparation of Nortis Kidney MPS

Kidney MPS devices were purchased from Nortis, Inc (Woodinville, WA). Device preparation and PTEC injections were performed by the investigators as previously reported^9^. PTEC MPS cultures were maintained serum-free in DMEM/F12 supplemented with 1x insulin-transferrin-selenium-sodium pyruvate, 50 nM hydrocortisone, and 1x Antibiotic-Antimycotic. For all experiments run for EVT as well as for CRS-17 and CRS-22, PTECs of passage 2 or lower were used from each individual donor kidney. For CRS-17 & CRS-22, PTECs were shipped on dry ice to the Kennedy Space Center laboratories. Following recovery from cryopreservation and expansion in 2D culture, MPS were seeded and allowed to culture as detailed in Fig. 3. As part of the EVT experimental design, a media cassette change was performed eight days after initiating KCPP flow.

#### Quantification of organ-specific injury biomarker KIM-1

DuoSet© ELISA kits were used to quantify human KIM-1 (R&D Systems, Minneapolis, MN, Cat. # DY1780B) in PT-MPS effluents following the manufacturer’s protocol. In brief 50-100 µL of effluent were tested in duplicates and concentrations determined based on the standard curves generated from manufacturer-supplied controls.

#### RNAseq data generation and analysis

To collect RNA samples from PTEC tubules, the PT-MPS devices were flushed with a volume of 1 mL RLT buffer (Qiagen, #79216) delivered through the abluminal inlet using a 1 mL slip-tip syringe (BD, #309659) equipped with a 22-gauge needle (BD, #305142) and collected at the outlet port. The RNA samples in RLT buffer were stored at −80^ o^C until extraction as described by Lidberg et al. ^11^.

### Preparation of the KCPP for space flight and ground control experiments

All KCPP components were prepared in BioServe laboratories and shipped to the launch site for final science integration and loading. In order to support both flight and ground control experiments, 8 perfusion pumps, 16 MPS housings, 16 valve blocks, 16 launch media cassettes, 16 treatment media cassettes, 16 fixative cassettes, two power modules, and two launch lockers with foam inserts were required. At the launch site laboratory facilities, at approximately 5 days before the launch, all cassettes were loaded with the corresponding reagent. This included launch media, treatment media, formalin, and RNAlater for the first flight and launch media, treatment media, and RNAlater for the second flight. The media, treatment, and fixative cassettes required for both the space flight experiment and the ground controls were loaded at the same time. Once loaded, they were placed at appropriate temperature control (4 °C or ambient) until use. At approximately 36 hours before launch the MPS chips for the space flight experiment were integrated into the MPS housings. Ground controls were loaded approximately 24 hours later. After the integration of the chips into the MPS housings, the valve blocks were connected and sealed to the MPS housings. Following this step, the entire housing/valve block assembly was connected to the launch media cassette and integrated into the perfusion pump. Each pump was powered and the system purged to clear any air bubbles potentially present from connecting the media cassette to the MPS housing/valve block assembly. Following the purge, the KCPPs were commanded to run mode. The KCPPs remained in this configuration for two hours to ensure the nominal operation of the KCPP. Once the nominal operation was confirmed, the four KCPPs were integrated into the launch locker with the power module and powered via an external power supply. The fully loaded locker was then turned over to NASA for loading into the launch capsule. The unit remained fully powered except during transfer from the external power source to the capsule power and when transferred from the launch capsule to the 2 incubators on orbit. After the KCPP reached the ISS, the crew powered down the locker, removed the 4 KCPP units from the launch locker, and loaded two KCPP and one power module each into two BioServe Space Automated Bioprocessing Laboratory (SABL) that operate on board the ISS. Ground controls followed the same process on a 24-hour delay but standard laboratory incubators were used to provide temperature and 5% CO_2_.

### Supplementary information

Supplementary Information

## Data Availability

Data generated and analyzed for this study are available from the corresponding author upon reasonable request.
