# Supplementary material for: Development of a kidney microphysiological system hardware platform for microgravity studies
Source: NPJ Microgravity. 2024 May 11;10:54. doi: 10.1038/s41526-024-00398-0 (PMC11088639; doi:10.1038/s41526-024-00398-0)
Supplement: Supplementary file 1 — Supplementary Information [file 41526_2024_398_MOESM1_ESM.pdf]

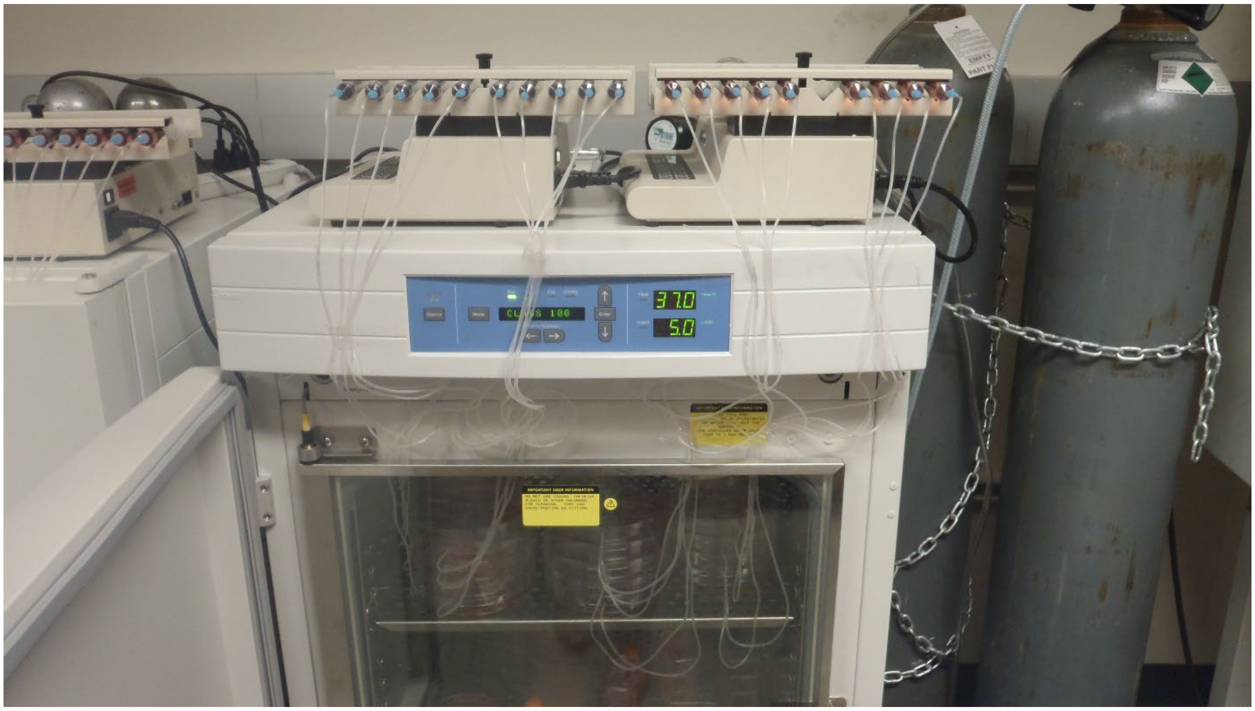

Supplementary Figure 1. Space requirements of PT-MPS using conventional syringe pumps. The footprint of each syringe pump necessitates a maximum of two pumps per tissue culture incubator and approximately 1 meter of tubing to connect media-containing syringes to each individual tubule of the PT-MPS.
